# The Meteoritics Trial: efficacy of methotrexate after remission-induction with tocilizumab and glucocorticoids in giant cell arteritis—study protocol for a randomized, double-blind, placebo-controlled, parallel-group phase II study

**DOI:** 10.1186/s13063-024-07905-4

**Published:** 2024-01-15

**Authors:** Lena Kreis, Christian Dejaco, Wolfgang Andreas Schmidt, Robert Németh, Nils Venhoff, Valentin Sebastian Schäfer

**Affiliations:** 1 Department of Internal Medicine III, Oncology, Haematology, Rheumatology and Clinical Immunology, University Hospital Bonn, Venusberg-Campus 1, 53127 Bonn, Nordrhein-Westfalen Germany; 2 Department of Rheumatology, Medical University Graz, Auenbruggerplaz 15, 8036 Graz, Austria; 3 Medical Centre for Rheumatology Berlin-Buch, Immanuel Krankenhaus Berlin, Lindenberger Weg 19, 13125 Berlin, Germany; 4 Institute of Medical Biometry, Informatics and Epidemiology, University Hospital Bonn, Venusberg-Campus 1, 53127 Bonn, Germany; 5 Department of Rheumatology and Clinical Immunology, Medical Center - University of Freiburg, Faculty of Medicine, University of Freiburg, Hugstetterstraße 55, 79106 Freiburg, Germany

**Keywords:** Giant cell arteritis, Methotrexate, Tocilizumab, Glucocorticoids, Remission maintenance, Vasculitis, Rheumatic disease, Ultrasound, Phase II trial

## Abstract

**Background:**

Glucocorticoids (GC) are the standard treatment for giant cell arteritis (GCA), even though they are associated with adverse side effects and high relapse rates. Tocilizumab (TCZ), an interleukin-6 receptor antagonist, has shown promise in sustaining remission and reducing the cumulative GC dosage, but it increases the risk of infections and is expensive. After discontinuation of TCZ, only about half of patients remain in remission. Additionally, only few studies have been conducted looking at remission maintenance, highlighting the need for alternative strategies to maintain remission in GCA. Methotrexate (MTX) has been shown to significantly decrease the risk of relapse in new-onset GCA and is already a proven safe drug in many rheumatologic diseases.

**Methods:**

This study aims to evaluate the efficacy and safety of MTX in maintaining remission in patients with GCA who have previously been treated with GC and at least 6 months with TCZ. We hypothesize that MTX can maintain remission in GCA patients, who have achieved stable remission after treatment with GC and TCZ, and prevent the occurrence of relapses. The study design is a monocentric, randomized, double-blind, placebo-controlled, parallel-group phase II trial randomizing 40 GCA patients 1:1 into a MTX or placebo arm. Patients will receive 17.5 mg MTX/matching placebo weekly by subcutaneous injection for 12 months, with the possibility of dose reduction if clinically needed. A 6-month follow-up will take place. The primary endpoint is the time to first relapse in the MTX group versus placebo during the 12-month treatment period. Secondary outcomes include patient- and investigator-reported outcomes and laboratory findings, as well as the prevalence of aortitis, number of vasculitic vessels, and change in intima-media thickness during the study.

**Discussion:**

This is the first clinical trial evaluating remission maintenance of GCA with MTX after a previous treatment cycle with TCZ. Following the discontinuation of TCZ in GCA, MTX could be a safe and inexpensive drug.

**Trial registration:**

ClinicalTrials.gov, NCT05623592. Registered on 21 November 2022.

EU Clinical Trials Register, 2022-501058-12-00.

German Clinical Trials Register DRKS00030571.

**Supplementary Information:**

The online version contains supplementary material available at 10.1186/s13063-024-07905-4.

## Introduction

### Background and rationale {6a}

Giant cell arteritis (GCA), affecting patients aged 50 years and older, is the most common form of systemic vasculitis. It manifests as granulomatous inflammation in branches of large vessels (LV) or LV themselves [1–4]. The pathogenesis of GCA involves the recruitment of T-cells and monocytes, leading mainly to intimal and medial proliferation that can reduce blood flow in affected arteries and result in (partial) ischemia. GCA may cause diffuse, temporal or occipital headache, scalp tenderness, or jaw claudication. One feared event is partial or complete vision loss due to anterior ischaemic optic neuropathy. Fever, weight loss, night sweats, and an intense acute-phase response are all symptoms of systemic inflammation [2]. The increased use of imaging for diagnosis, such as ultrasound, magnetic resonance imaging (MRI), or fluorodeoxyglucose positron emission tomography, has broadened the spectrum of diagnostic procedures, making temporal artery biopsy almost obsolete [1, 5].

The standard treatment for GCA consists of glucocorticoids (GC), which are effective in providing rapid symptom relief and prevent ischaemic complications if applied in a timely manner [6, 7]. However, short and long-term GC therapy is associated with significant adverse side effects, and the risk of relapse is high [7–10]. Therefore, GCA treatment requires other therapeutic agents. Several conventional synthetic disease-modifying anti-rheumatic drugs (DMARDs) and biological DMARDs have been investigated as agents sparing GC in GCA. Due to their limited benefits, toxicity or side effects, most of them were not recommended [6, 11]. A possible immunosuppressive drug to decrease the cumulative GC dose and sustain remission is the interleukin-6 receptor antagonist tocilizumab (TCZ). In a randomized, double-blind, placebo-controlled, phase II trial, the treatment of new onset or relapsing GCA with TCZ was addressed for the first time. Patients received oral tapered GCs added with TCZ or placebo infusions every 4 weeks. After 52 weeks of treatment, a relapse-free remission rate of 85% (17 out of 20) was observed in the verum group, compared to a rate of 20% (2 out of 10) in the placebo group. The 17 patients in complete GC-free remission of the TCZ group at week 52 discontinued TCZ treatment. After a mean of 29.3 months, it was observed that nine patients (53%) remained in remission, while eight (47%) experienced a relapse after a mean of 6,3 months [12, 13]. In the phase III GiACTA trial, subcutaneous (SC) TCZ every or every second week in combination with a 26-week GC tapering scheme was compared with a 26- or 52-week GC tapering scheme alone. After 52 weeks, sustained remission was observed in 56% and 53% of the TCZ groups that received it every and every second week. In the groups receiving GC, sustained remission was only seen in 14% and 18%. The cumulative GC doses were also reduced in patients treated with TCZ [14, 15]. In the second phase of the GiACTA study, 59 patients receiving weekly TCZ treatment and achieving glucocorticoid-free remission at week 52 underwent discontinuation of TCZ to observe relapse rates over a period of 104 weeks. During this phase, only 42% (25 out of 59) of the patients remained in glucocorticoid-free remission, while 58% failed to remain in remission. Among the patients who received TCZ every other week, only 29% (8 out of 28) achieved glucocorticoid-free remission after discontinuation of TCZ, while 71% (16 out of 28) experienced a relapse and failed to maintain remission [16].

The primary risk of treatment with TCZ is the increased risk of infections [14, 17, 18]. Moreover, TCZ therapy is significantly more expensive than GC therapy alone and results in C-reactive protein (CRP) not being used as an acute inflammatory marker in clinical practice [19, 20]. The optimal duration of TCZ treatment for patients in a stable remission without GC remains uncertain, as does the appropriate strategy following treatment discontinuation to minimize relapse rates. These findings highlight the need for alternative strategies to maintain remission in GCA, though trials investigating this are scarce.

The combination of methotrexate (MTX) and GC also effectively reduced the relapse rate and the cumulative GC doses in new or relapsing GCA. Currently, three randomized controlled trials (RCTs) accessing 7.5–15 mg MTX per os weekly for newly diagnosed GCA are available. Although only one of the three RCTS showed a significant positive effect in the reduction of one or more relapses and a reduction in cumulative GC doses [21–23], a meta-analysis by Mahr et al. [24] of these three RCTs with a total of 161 patients reported a positive effect of additive MTX: The risk reduction of first relapse was significant with a hazard ratio of 0.65. The number needed to treat to prevent one relapse was 3.6. A reduction in cumulative GC doses was also observed. A retrospective observational study also demonstrated a positive effect in reducing relapses [25]. MTX is a safe and well-established drug with an extensive history of use in many rheumatologic diseases [26].

### Objectives {7}

Our hypothesis is that MTX is able to maintain remission, once stable remission has been induced by GCs and TCZ and will prevent the occurrence of relapses. The primary objective of this study is to evaluate the efficacy of MTX in maintaining remission compared to a placebo after discontinuation of TCZ in patients in remission with GCA. The primary endpoint is the time to relapse. To test the efficacy of MTX, we will evaluate patient- and investigator-reported outcomes and laboratory findings. As secondary objectives, we will examine the prevalence of aortitis, the number of vasculitic vessels accessible by ultrasound and the change of intima-media thickness (IMT) during the study.

### Trial design {8}

**Fig. 1:**
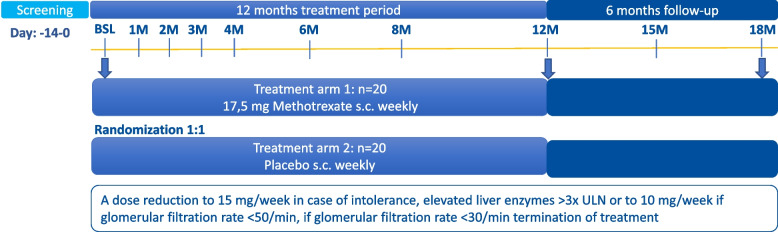
Study design. Arrows indicate the time at which the MRI is performed, BSL, baseline; M, month; mg, milligrams; ULN, upper limit of normal

This is a randomized, double-blind, placebo-controlled, parallel-group phase II study to estimate the efficacy and safety of MTX to maintain remission in GCA patients who have discontinued TCZ treatment while being in remission. Patients will be randomly assigned 1:1 into MTX (*n* = 20) and placebo (*n* = 20) arms. The study will comprise two phases, a 12-month treatment phase with weekly administration of study drug and a 6-month follow-up phase without study drug administration (see Fig. 1).

## Methods: participants, interventions and outcomes

### Study setting {9}

The study will be conducted as a monocentric study at the Department of Rheumatology and Clinical Immunology, Clinic of Internal Medicine III, University Hospital Bonn, Germany.

### Eligibility criteria {10}

Subjects will only be included in the study if they meet all of the following criteria:

1. Subjects male or female, aged ≥ 18 years
2. Written informed consent of the capable subject for voluntary participation in the study
3. Diagnosis of GCA as confirmed by the investigator fulfilment (also in retrospect) of the proposed extended 1990 classification criteria for GCA
4. Previous treatment with GC and TCZ for new or relapsing GCA
5. GCA patients who have been treated with TCZ 162 mg SC weekly and in whom discontinuation of TCZ therapy has been decided by the treating rheumatologist
6. Duration of TCZ therapy without concomitant MTX therapy for at least 6 months before inclusion
7. Patients be in stable remission (defined as the absence of signs and symptoms of GCA and CRP < 1 mg/dl), off GC for at least 1 month before the screening visit
8. Willing and able to inject MTX or placebo SC
9. Male and female subjects agreeing to conduct efficient contraception (unless they have no childbearing potential)

Subjects will not be included in the study if any of the following criteria apply:

1. Severe renal (glomerular filtration rate < 30/min) failure
2. Conditions other than GCA requiring continuous or intermittent treatment with oral or parenteral GCs unless the last exposure to GCs was > 1 month before screening
3. Other inflammatory rheumatic diseases (e.g. rheumatoid arthritis)
4. Current treatment with any other conventional, biologic or targeted synthetic DMARD except TCZ
5. Elevation of transaminases above three times the upper limit of normal according to local laboratory cut-off values
6. Simultaneous participation in another clinical trial, or participation in a clinical trial taking an investigational product, up to 30 days prior to participation in this clinical trial
7. Pregnant or breast-feeding women
8. Contraindications for therapy with methotrexate (metex®), as indicated in the summary of product characteristics

### Who will take informed consent? {26a}

After weighing up the individual risks and benefits for patients, informed consent will be obtained from patients by the treating specialist in internal medicine and rheumatology.

### Additional consent provisions for collection and use of participant data and biological specimens {26b}

In addition, patients will be requested to give informed consent for additional blood samples to be stored for possible future research.

## Interventions

### Explanation for the choice of comparators {6b}

Currently, MTX is used as an alternative for TCZ for patients with refractory or relapsing GCA, or in the presence of or increased risk of GC-associated sequelae, as recommended by the EULAR and ACR guidelines [27, 28]. The most critical side effects of MTX therapy include gastrointestinal symptoms, hepatotoxicity, mucocutaneous reactions, increased risk of infections, and MTX pneumonitis [26]. Enquiring about possible symptoms and assessing transaminases and kidney function will ensure safe monitoring and adjustment of treatment if necessary.

We hypothesize that MTX is effective and safe in maintaining remission once GC and TCZ have induced stable remission and will prevent the occurrence of relapses and GC-related side effects.

Following the discontinuation of TCZ, most relapses occur within the first 5 months [13]. Therefore, a 12-month period appears adequate to assess the difference between the MTX and placebo groups.

### Intervention description {11a}

The last TCZ administration should be 7–12 days before the first study drug administration. Patients will self-administer SC injections of either a 17.5 mg MTX syringe or a matching 0.9% NaCl-solution placebo syringe once weekly, along with 5 mg of oral folic acid 24 h after injection to mitigate side effects. Consumption of alcohol on the day of the MTX or placebo injection should be avoided. If a dose is missed, it can be injected within 2 days. The duration of drug intake will be 12 months.

**Fig. 2:**
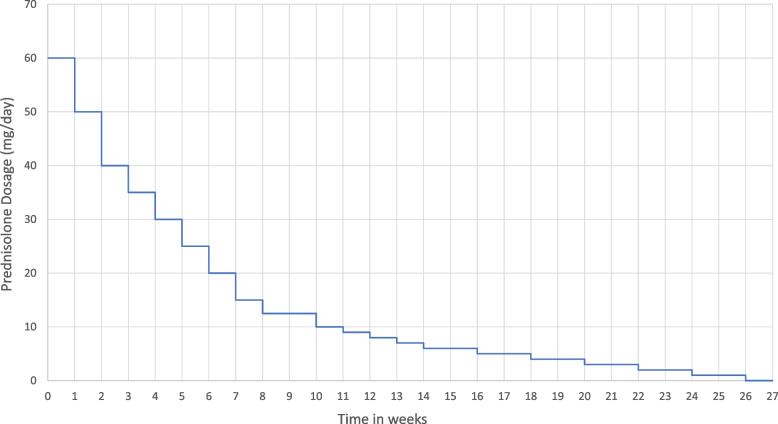
Prednisolone tapering regime in case of relapse. mg, milligrams

In the event of relapse, the treating rheumatologist will be able to prescribe a prednisolone dose determined after clinical evaluation, which will be tapered according to the GIACTA prednisolone tapering regime (Fig. 2) [14]. If ischaemic symptoms (e.g. of the eye) appear, patients will receive intravenous (IV) methylprednisolone at a dosage of 500–1000 mg for five days. The administration of the study drug will be continued at the same dose in case of relapse. If more than three relapses occur, study treatment will be terminated, and patients will be treated at the discretion of the investigator.

### Criteria for discontinuing or modifying allocated interventions {11b}

A dose reduction to 15 mg/week in case of intolerance, elevated liver enzymes > 3 × upper limit of normal or to 10 mg/week for glomerular filtration rate < 50/min will be possible. Treatment will be terminated if the glomerular filtration rate falls below 30/min.

### Strategies to improve adherence to interventions {11c}

Patients will receive a box with the easy-to-use prefilled syringes, including a reserve one every visit. At baseline (day 0), there will be an instruction on administering the syringes concerning injection technique, date, location and documentation in a patient diary. The first self-injection will take place under supervision. The patient diary and the boxes with any unused syringes will be returned at the next visit to verify the correct dates, locations of injections and the number of unused syringes.

### Relevant concomitant care permitted or prohibited during the trial {11d}

No concomitant care is prohibited as long as it is documented in the case report form (CRF).

### Provisions for post-trial care {30}

Patients will be monitored for the occurrence of adverse events throughout the 6-month follow-up period after the treatment period. All study participants are covered by insurance for damages caused by the administered study drugs.

### Outcomes {12}

#### Primary outcome

##### Assessment of efficacy of MTX on sustained remission

The primary outcome is the prevalence of sustained remission in both groups measured by time to relapse during the 12-month treatment period. Remission is defined as the absence of relapse and the normalization of the CRP concentration to less than 10 mg/L. Relapse, defined as the recurrence of signs or symptoms of GCA or as an elevation of CRP of 10 mg/L or more attributable to GCA, is determined using the GCA relapse assessment as judged by the treating rheumatologist.

### Secondary outcome

#### Assessment of the need for rescue therapy with prednisone

A secondary outcome is to assess the need for rescue therapy with prednisone through cumulative prednisone doses at months 6, 12, and 18.

#### Assessment of the number of relapses during the study

Moreover, there will be a measurement of the number of relapses per patient during the 12-month treatment period, the time to first, second and third relapse after randomization, and the percentage of patients with a relapse until month 6 and until month 18 after discontinuation of TCZ.

#### Evaluation of the impact of MTX maintenance therapy on patient-reported outcomes and investigator-reported outcomes

Another secondary outcome includes assessing health-related quality of life (HRQoL) using the Short-Form-36 (SF-36) questionnaire. We will use the Functional Assessment of Chronic Illness Therapy–Fatigue Scale (FACIT-F) questionnaire to measure self-reported fatigue. The impact of MTX on patient- and investigator-reported outcomes will be evaluated through the use of Patient Global Assessment of disease activity (PGA), Patient Assessment of Pain (PAP), and investigator-reported Evaluator Global Assessment of disease activity (EGA).

#### Assessment of the vasculitic involvement and change of IMT in patients with/without relapses and the influence of study treatment on patients with aortitis

With the utilization of ultrasound examination at every study visit and the MRI at baseline, months 12 and 18, we will evaluate the number of vasculitic vessels, change of IMT and the prevalence of aortitis.

#### Assessment of the impact of MTX on the visual symptoms and other ischaemic complications related to GCA

Another secondary outcome involves assessing the impact of MTX maintenance therapy on visual symptoms and other ischaemic complications related to GCA measured with the occurrence of symptoms and signs related to GCA.

#### Influence of study treatment on inflammation

In addition, the impact of study treatment on inflammation will be assessed by the proportion of subjects with elevated erythrocyte sedimentation rate (ESR) and C-reactive protein (CRP) levels.

#### Evaluation of the safety of MTX

Moreover, the safety of MTX will be measured by monitoring the occurrence of adverse events and serious adverse events, and the incidence of GC-related adverse events will be assessed.

### Participant timeline {13}

After the screening, eight study visits will occur during the 12-month treatment and two during the 6-month follow-up. Patient- and investigator-reported outcomes, laboratory assessments and ultrasound examination of potentially by GCA affected arteries will be assessed at every study visit (see Table 1 for details).

**Table 1:** Schedule of activities

**Visit**		**1**	**2**	**3**	**4**	**5**	**6**	**7**	**8**	**9**	**10**	
**Timepoint**	***Day: -14–0***	**Day 0**	***Month 1***	***Month 2***	***Month 3***	***Month 4***	***Month 6***	***Month 8***	***Month 12***	***Month 15***	***Month 18***	***At any time during the study***^**b**^
	**Screening**	**Baseline**	**Treatment Period**							**Follow-up**		**Relapse Visit**
**Written Informed Consent**	X											
**Demography and medical history**	X											
**Inclusion/Exclusion Criteria**	X	X										
**Randomization**	X	X^a^										
**Administration of study drug**		X	X	X	X	X	X	X	X			
***Concomitant Medications***	X	X	X	X	X	X	X	X	X	X	X	X
**Safety Assessments**												
***Physical Examination***	X	X							X	X	X	X
**Vital Signs**	X	X	X	X	X	X	X	X	X	X	X	X
***12-lead ECG***	X											
***Adverse Events***		X	X	X	X	X	X	X	X	X	X	X
***Efficacy Assessments***												
***GCA remission assessment, GCA relapse assessment, Patient global assessment, Patient pain assessment, Evaluator Global Assessment of disease activity***	X	X	X	X	X	X	X	X	X	X	X	X
***FACIT- Fatigue, SF-36***		X							X	X	X	
***BVAS***		X	X	X	X	X	X	X	X	X	X	X
***Magnetic resonance imaging***		X							X		X	
***Laboratory Assessments***												
***Serum Chemistry, Hematology, CRP, ESR***	X	X	X	X	X	X	X	X	X	X	X	X
***Pregnancy Test***^**c**^	X^d^	X^e^	X^e^	X^e^	X^e^	X^e^	X^e^	X^e^	X^e^	X^e^	X^e^	X^e^
***Hepatitis B and C testing***	X											
***Imaging***												
***Ultrasound examination***		X	X	X	X	X	X	X	X	X	X	X
***Magnetic resonance imaging***		X							X		X	

*ECG* Electrocardiography, *GCA* Giant cell arteritis, *CRP* C-reactive protein, *ESR* Erythrocyte sedimentation rate, *FACIT-Fatigue* Functional Assessment of Chronic Illness Therapy – Fatigue Scale, *SF-36* Short Form-36, *BVAS* Birmingham Vasculitis Activity Score

^a^If not already occurred in the screening phase

^b^Relapse visits can be made at any time during the study

^c^For women of childbearing potential

^d^serum β-HCG level

^e^urine β-HCG level

### Sample size {14}

According to the exploratory character of the trial, the main aim is more to estimate the effect size rather than perform confirmatory testing. Therefore, the half width of the 95% confidence interval (as a measure of precision of the estimate) was used as the bases for the sample size calculation. Assuming relapse rates of 40% and 60% for the verum and placebo groups, respectively, with a sample size of 20 subjects per group (meaning expected numbers of events of 8 and 12 in the two groups), precision of approximately 0.9 for the log-transformed hazard ratio will be achieved. We take into consideration the relapse rates observed after discontinuation of SC TCZ from GiACTA extension, which were 58% in the weekly TCZ group and 71% in the biweekly group [16]. Therefore, we assume a relapse rate of 60% for the placebo group. Considering a 30% drop-out rate, it is estimated that 52 subjects need to be enrolled to have 40 evaluable subjects for analysis.

### Recruitment {15}

Enrolment started in November 2022, with an anticipated recruitment duration of 1.5 years, concluding in May 2024. Currently, the Rheumatology and Clinical Immunology Clinic of Internal Medicine III at the University Hospital Bonn is treating more than 360 patients with GCA, 180 of whom receive TCZ therapy. The recruitment for this purpose is expected to be completed before May 2024.

## Assignment of interventions: allocation

### Sequence generation {16a}

After enrollment by the study centre, the patients will be assigned a study number through the electronic case report form (eCRF) system “XClinical”. Randomization will take place via an interactive web response system. Appropriate kit lists will be generated by a biometrician not involved in the data analysis. The eCRF system will assign kits with the selected dose regimen based on the generated kit lists for each visit. Both the patients and the investigator or sponsor will be unable to view the treatment allocation following randomization.

### Concealment mechanism {16b}

See above.

### Implementation {16c}

See above.

## Assignment of interventions: blinding

### Who will be blinded {17a}

Participants, care providers, study management and data analysts will be blinded. The pharmacy, using the kit lists, will prepare the syringes. By masking the plunger with tape to conceal the colour, the syringes’ identical appearance ensures that distinguishing between MTX and placebo is not possible.

### Procedure for unblinding if needed {17b}

Emergency codes for unblinding (providing information about the administered drug) are prepared by the corresponding pharmacy, sealed in emergency envelopes, and stored in a known location accessible to all study personnel. The monitor will control the integrity of envelopes. Unblinding may be necessary to ensure patient safety in the event of ischaemic symptoms of the eye, the need for future treatment dependent on the knowledge of group type, accidental administration of study medication, death with a causal relationship to study treatment, serious adverse event (SAE) or suspected unexpected serious adverse reaction potentially related to study medication.

## Data collection and management

### Plans for assessment and collection of outcomes {18a}

All assessments and outcome measures are shown in Table 1.

Data collection will consist of each participant’s medical history, including past and current conditions, treatments, surgeries, and current medications, as well as their year of birth, age, sex, height, weight, and body mass index. During each study visit, a rheumatology specialist will evaluate whether patients are in remission or have experienced a relapse. The patients will be asked about the general effects of GCA in the PGA, and about the pain associated with GCA in the PAP. The investigator will rate the impact based on the patient’s responses on a scale of 0 to 100, where 0 indicates no impact, and 100 indicates the worst possible impact. The investigator will assess the patient’s overall GCA-related condition using the EGA, ranging from 0 to 100, with 0 indicating no impact and 100 indicating the worst possible impact. We will assess HRQoL in eight health concepts using the German version of the SF-36 (version 2) questionnaire at baseline, month 12, and twice during follow-up. The questionnaire contains 36 questions that generate a score between 0 and 100, where 0 represents maximal impairment, and 100 indicates no impairment [29]. The FACIT-F questionnaire will be utilized to measure the fatigue experienced by GCA patients and to assess the changes in fatigue due to treatment throughout the study. It consists of 13 items, each assigned a numerical score from 0 to 52, with higher scores indicating less fatigue. This questionnaire has been validated previously for various rheumatic diseases [30]. The Birmingham Vasculitis Activity Score (version 3) is a validated tool for assessing systemic vasculitis, considering nine sections potentially affected by systemic vasculitis. Points are assigned to each abnormality due to active vasculitis, with higher scores indicating new or improved status and lower scores indicating persistent status. The score is demonstrably repeatable, reproducible and sensitive to changes in disease status [31]. Due to the fact that most sections do not cover the manifestations of GCA and it is possible to have a score of 0 within active GCA, its utility in GCA is limited. Consequently, caution must be exercised when evaluating GCA using it [32].

Laboratory testing will enable the measurement of haemoglobin, haematocrit, red blood cell count, MCV, MCH, MCHC, white blood cell count, lymphocytes, and platelet count. Serum chemistry accesses urea, uric acid, creatinine, glucose, potassium, sodium, chloride, calcium, total protein, albumin, creatine kinase, total fasting cholesterol, LDL, AST, ALT, alkaline phosphatase, and total bilirubin. To examine inflammation CRP and ESR will be determined.

The use of ultrasound for diagnosis shows high sensitivity and specificity in accessing the halo sign (homogeneous, hypoechoic wall thickening), compression sign (vessel wall still visible under complete compression) and measurement of IMT to distinguish between normal or vasculitic vessels. There are cut-off values for IMT to distinguish between normal and vasculitic arteries [33–36]. Ultrasound examination of the following arteries will enable the measurement, collection, and evaluation of changes in intima-media thickness (IMT), the number of vasculitic vessels defined by the OMERACT ultrasound definitions, and the corresponding cut-off values at each study visit [33, 36–38]: the superficial common temporal artery with frontal and parietal branches, the facial artery, the common carotid artery, the vertebral artery, the axillary artery, the brachial artery and the subclavian artery. Measuring the IMT of the common temporal artery with its frontal and parietal branches as well as the axillary artery on both sides allows the calculation of the OMERCAT GCA Ultrasonography Score (OGUS). This score was developed to monitor disease activity and ultrasound manifestation of GCA, especially in clinical trials. The calculation involves dividing the IMT values of each artery by the rounded cut-off value and summing these values together. The resulting sum is then divided by the number of arteries measured. A value ranging from 0 to 1 is considered within the normal range, while a value above 1 indicates an abnormality [39]. Furthermore, a transorbital ultrasound of the central retinal artery will be used to measure peak systolic velocity and end diastolic velocity and calculate the resistance index.

Using MRI, we will investigate the aorta to detect aortitis or aortic complications at baseline, after 12 months of treatment and 6 months after discontinuation of the study drug.

### Plans to promote participant retention and complete follow-up {18b}

The accessibility of the study personnel, available to address participant enquiries related to side effects, symptoms of GCA, or general questions, along with the short intervals between study visits, will enable increased participant retention. If necessary, relapse visits will take place as soon as possible to treat relapse quickly and adequately. Participants will be able to withdraw from the trial at any time without disadvantages. The decision for withdrawal could also be made at the investigator’s discretion for safety, behavioural, or administrative reasons. Subjects who withdraw from the trial before randomization will be listed, including the reason for withdrawal. Subjects who drop out after randomization will be analysed using all available data. They will be asked to attend the end-of-study visit (Visit 10).

### Data management {19}

Data collection will be performed using paper-based methods and then transferred electronically into the eCRF using the MARVIN software provided by XClinical. Only authorized trial personnel will have permission to promptly enter all these data into the CRF. The program will automatically document all data entries and corrections on the eCRF pages through an “audit trail” feature. The investigator will sign the completed data electronically. The monitor will be responsible for verifying the eCRF at regular intervals throughout the trial to verify the adherence to the protocol and the completeness, accuracy, and consistency of the data.

### Confidentiality {27}

To safeguard pseudonymity, patients will be assigned an identification number for the study. The subject identification list, in which the investigator will have to record the trial participation of each subject, will be stored on the study side. This list enables the identification of each subject and contains the subject number, name, birth date and the date of inclusion of the subject into the trial. All analysed data will be evaluated exclusively based on the identification number, ensuring complete confidentiality of patient information.

### Plans for collection, laboratory evaluation and storage of biological specimens for genetic or molecular analysis in this trial/future use {33}

For further research, 20 ml of serum will be collected at every study visit, sent to Biobank Bonn and stored at – 80 °C.

## Statistical methods

### Statistical methods for primary and secondary outcomes {20a}

As this is the first study with MTX after TCZ in GCA, a detailed analytical and graphical data exploration will take place to characterize the effect of MTX in the given indication.

The primary objective of the trial (to assess the efficacy of MTX in maintaining remission after discontinuation of TCZ in patients in remission with GCA) will be addressed by applying a treatment policy strategy for the primary estimand:

The target population consists of GCA patients who have discontinued TCZ treatment while being in remission (see also in- and exclusion criteria) independently of the occurrence of any intercurrent events.

The variable of interest will be the time to the occurrence of the first relapse during the 12-month treatment period. The population level summary will be the Kaplan–Meier estimate of the relapse time.

The treatment will be self-administered SC injections of either 17.5 mg of MTX syringe or a matching 0.9% NaCl-solution placebo syringe once weekly (see intervention section for details).

Kaplan–Meier estimators will be used to summarize the time to first relapse, including the median, 25th, and 75th percentiles (where feasible) and 95% confidence interval for the median being. The hazard ratio (together with the corresponding 95% confidence interval) will be estimated by a Cox regression model with the treatment as the sole explanatory variable.

The main population of any further summaries and analyses will be the intention-to-treat population. Additional analysis will be performed in the per-protocol population, comprising patients with sufficient protocol adherence.

For secondary outcomes such as the time to second and third relapse, descriptive statistics will be used. Repeated relapses will be handled by models developed for recurrent events [40, 41] depending on the number of relapses. Proportions will be compared (in a purely descriptive manner) using Fisher’s exact test. All parameters will be summarized by descriptive statistics and will be presented in corresponding tables and figures as applicable. Repeatedly measured continuous variables (like, e.g. the inflammatory laboratory parameters) will be analysed by corresponding mixed effects models for repeated measures (with patient as the random effect) to estimate the effect of MTX and to characterize the time course of the parameters. Outcomes of a discrete nature (like, e.g. the number of relapses) will be submitted to corresponding regression models (e.g. Poisson regression) to calculate the effect estimate of MTX.

The number of subjects with adverse events and the number of adverse events will be summarized by treatment with separate summaries for GC-related adverse events.

### Interim analyses {21b}

Not applicable, as no interim analyses are planned.

### Methods for additional analyses (e.g. subgroup analyses) {20b}

Additional outcomes (like, e.g. BVAS) will be summarized by descriptive statistics. The differences between the treatment groups will be estimated using similar methods as described for the secondary outcomes.

### Methods in analysis to handle protocol non-adherence and any statistical methods to handle missing data {20c}

During the failure time analysis, subjects with premature termination will be censored at the time when they leave the trial. Missing values for the analysis of the secondary endpoints will be treated as such. A sensitivity analysis will be performed using likelihood-based methods (like logistic regression or mixed linear models). The pattern and number of missing values in the two treatment groups will be shown in corresponding summaries.

### Plans to give access to the full protocol, participant-level data and statistical code {31c}

We plan to share data if an adequate proposal is submitted.

## Oversight and monitoring

### Composition of the coordinating centre and trial steering committee {5d}

This is a monocentric study of the Department of Rheumatology and Clinical Immunology, Clinic of Internal Medicine III, University Hospital Bonn, Germany and the head of rheumatology (VSS) is the principal investigator. The coordinating centre, data management, monitoring and statistical analysis will be performed by the Clinical Study Core Unit of the Study Center Bonn.

### Composition of the data monitoring committee, its role and reporting structure {21a}

The Data Safety Monitoring Board (DSMB) is an independent committee monitoring the study progress of the safety of trial participants and the quality of the collected data (by monitoring reports). Its role includes making recommendations regarding the trial’s discontinuation, modification or continuation. The DSMB’s main responsibility in this study is to review all SAEs reported by patients, assess any unmonitored data, and suggest modifications to the trial. The DSMB includes three members with long-term experience in conducting studies and individual expertise in the field of GCA and biometrics. The DSMB will meet at least once per year for the duration of the clinical trial, or once 50% of the patients have been recruited, for a total of three times.

### Adverse event reporting and harms {22}

(Serious) adverse events recorded by the patient or by the study personnel will be recorded and followed until resolving or stabilizing. While documentation in source and eCRF, it is important that the investigator evaluate the event for intensity, seriousness and causality. For SAEs, a second evaluation by the sponsor for causality and expectability, with safety data available only to the sponsor will occur. Every SAE has to be reported immediately to the sponsor, principal investigator and study coordinating centre.

### Frequency and plans for auditing trial conduct {23}

Monitoring visits will occur six times during the trial to ensure consistent, complete, and reliable data. This trial may be selected for audit by sponsor representatives or for inspection by site-responsible representatives of the local regulatory authority.

### Plans for communicating important protocol amendments to relevant parties (e.g. trial participants, ethical committees) {25}

The clinical trial protocol and its amendments must be approved by the appropriate regulatory authorities. In addition subject information and informed consent and any other written information provided to subjects must be approved by the respective principal research ethics committee.

### Dissemination plans {31a}

In collaboration with all participating institutions, the coordinating investigator will disseminate the trial’s findings through publication in peer-reviewed journals and conference presentations.

Individual participant data (including data dictionaries) that underlie results concerning primary or secondary endpoints reported in a published scientific article will be shared on demand after de-identification. Furthermore, the study protocol and informed consent form will be made available. The data will be shared after a scientific proposal has been submitted.

## Discussion

With the design of a placebo-controlled double-blind study, we aim to evaluate if MTX is effective for remission maintenance after discontinuation of TCZ in GCA. Despite the current clinical use of MTX for the treatment of GCA, there is still a need to improve the available evidence and expand the knowledge base regarding the ability of this drug to prevent a relapse of patients in stable remission. Notably, this study is the first to evaluate a therapeutic agent following TCZ and GC in GCA patients in stable remission. De-escalating therapy to less potent therapeutic regimes after stable remission has been achieved is also a proven strategy in other vasculitides. In ANCA-associated vasculitis in which remission has been induced with GC and cyclophosphamide, cyclophosphamide should be replaced with azathioprine, MTX or rituximab to maintain remission and avoid the toxicity of long-term cyclophosphamide [42, 43]. Furthermore, the significance of this study is heightened since TCZ is currently the only approved biological agent for treating GCA, with no other biological agent having demonstrated equal efficacy in GCA treatment thus far.

Studies included in the meta-analysis by Mahr et al. investigated MTX only at maximum doses of 7.5 mg-15 mg (mean 11.1 mg/week) with oral administration, and further, investigated the value of this drug only in new-onset or relapsing GCA patients [21, 23–25]. In the present study, we decided to use a starting dose of 17.5 mg/week, due to expert opinion, with the flexibility to decrease the dosage to 15 mg/week or 10 mg/week in the event of intolerance, elevated transaminases or reduced GFR. This approach ensures a high dose of MTX within the context of GCA while also maintaining patient safety and quality of life and reducing drop-out rates. In our study, targeting an elderly population potentially affected by renal impairment, we have deliberately set the methotrexate (MTX) dose at 17.5 mg/week, which is below the maximum possible dose. This cautious approach is designed to reduce the risk of adverse effects associated with increased MTX plasma concentrations, a concern particularly pertinent in individuals with compromised renal function. As the first trial for MTX in GCA we decide to use SC instead of oral application. It is known that the bioavailability of SC MTX is greater than that of oral administration. The area under the curve (AUC) of plasma concentrations in the first 24 h after application is higher for SC MTX application, and especially for doses ≥ 15 mg the mean AUC plateaued with oral administration, while SC administration leads to dose-proportional manner [44, 45]. In other inflammatory diseases, e.g. rheumatoid arthritis, SC administration has demonstrated greater efficacy and fewer treatment failures compared to oral administration, while there is no difference in tolerability [46, 47]. By analogy, one can also assume similar effects with the GCA. In addition, the SC administration should not cause compliance problems in the current trial, as the patients have already performed SC applications during TCZ therapy.

One great strength of this study is the additional data provided by imaging techniques. By using MRI before, after 12 months of study treatment and 6 months after treatment discontinuation, we will investigate the proportion of (silent) aortitis demonstrating LV involvement after TCZ treatment and the influence of study medication on it. Since the EULAR recommends using ultrasound examination of the temporal and axillary arteries as the first imaging modality [48], the diagnosis of GCA mainly focuses on these arteries, making data on the presence of aortitis not regularly obtained. Aortitis as LV involvement seems to be a predictor for decreased survival and a risk factor for relapse [49, 50]. The additonal MRI examinations will allow us to include the data about the presence of aortitis according to relapse to our analysis. Additionally, patients with GCA are known to have a higher incidence of aortic complications such as aneurysms and aortic dilatation, particularly in the thoracic aorta [48, 49, 51–53]. There is a scarcity of evidence-based knowledge on specifically monitoring and treating these complications. Hence, gathering more data to make recommendations is crucial, particularly in the long run.

Ultrasound findings are used as a monitoring tool in GCA, but there are few data from RCTs showing changes, especially in relation to the use of study drugs. The PROTEA trial demonstrated a rapid reduction in (the sum and maximal) IMT of the halo sign in temporal arteries with standard GC treatment, while the reduction in axillary arteries was delayed. In cases of relapse, 94.1% of ultrasound examinations showed a halo sign with higher IMT compared to pre-relapse examinations [54]. The change in ultrasound findings during GC treatment was also observed using the OGUS score, which was tested on PROTEA data and demonstrated a large and very large sensitivity to change [39]. The changes in IMT observed during a 3-day treatment with IV pulse GC followed by TCZ monotherapy in the GUSTO trial also indicate the efficacy of TCZ treatment in GCA. During the initial 3-day IV GC treatment, there was a sharp decrease in IMT of the temporal artery, followed by an increase until week 4, and a subsequent continuous decrease in IMT during TCZ monotherapy until week 52. Decreasing of IMT in the axillary and subclavian arteries was smaller, delayed, and reached a plateau [55]. In conclusion, it is established that the IMT of the temporal artery decreases during therapy with GC and TCZ and may increase in the event of a relapse, wherein the effect in LV, such as axillary and subclavian artery, is reduced. Additional data provided by ultrasound examination ten times during the present study will gain more information about the effectiveness of MTX and the influence on IMT and the number of vasculitic vessels during the study. An interesting feature of the ultrasound examination is to use the additional information from monitoring of ultrasound pathologies and the potential changes of ultrasound findings as a predictor of future relapse.

A limitation of this trial is the low sample size and the monocentric design. Since this is the first study addressing MTX as a potential therapeutic agent after discontinuation of TCZ, it is the first step to estimate the effect size. Further (multicentric) studies with larger sample sizes and similar study designs are needed to improve the statistical accuracy.

MTX, as a less potent immunosuppressive drug compared to TCZ, significantly decreases the risk for infections. It is a safe, inexpensive agent with extensive experience of use by rheumatologists. In the case of effectiveness, it could be a possible way to treat non-active GCA after TCZ in the long term, even for elderly patients with multiple comorbidities.

## Trial status

The protocol version number is 4.0 dated 21 July 2022. The recruitment began on 23 November 2022 and is scheduled to be completed by 23 May 2024.

### Supplementary Information

**Additional file 1.**

## Data Availability

We plan to share data if an adequate proposal is submitted.

## Abbreviations

AUC: Area under the curve
CRF: Case report form
CRP: C-reactive protein
DMARD: Disease-modifying anti-rheumatic drug
DSMB: Data Safety Monitoring Board
eCRF: Electronical case report form
EGA: Investigator reported Evaluator Global Assessment of disease activity
ESR: Erythrocyte sedimentation rate
FACIT-F: Functional Assessment of Chronic Illness Therapy – Fatigue Scale
GC: Glucocorticoids
GCA: Giant cell arteritis
HRQoL: Health-related quality of life
IMT: Intima media thickness
IV: Intravenous
LV: Large vessels
MRI: Magnetic resonance imaging
MTX: Methotrexate
OGUS: OMERACT GCA Ultrasonography Score
PAP: Patient Assessment of Pain
PGA: Patient Global Assessment of disease activity
RCT: Randomized controlled trial
SAE: Serious adverse event
SC: Subcutaneous
SF-36: Short-Form 36
TCZ: Tocilizumab
